# Characterization of MMP-9 and -12 KO mice in a model of acute smoke exposure

**DOI:** 10.1186/1476-9255-10-S1-P26

**Published:** 2013-08-14

**Authors:** Franklin J Schlerman, John E Kubera, Stephen P Pazdziorko, Michael R Bowman, Bruce A Jacobson, Margaret Fleming, Cara MM Williams, Joseph P Sypek

**Affiliations:** 1 Inflammation and Remodeling Research, Pfizer Cambridge Massachusetts, 02140, USA

## 

Short-term cigarette smoke exposure in mice has been shown to exhibit some similarities to cell and cell-mediated inflammation seen in COPD patients. We sought to identify biomarkers in the lungs and bronchoalveolar lavage fluid (BALF) that would characterize this inflammation. Mice were exposed; nose only, to 600 mg/m^3^ of cigarette smoke from 2R4F Kentucky Reference cigarettes twice daily for three consecutive days. Sham smoked animals were exposed to room air. Short-term cigarette smoke exposure induced a mild but statistically significant increase in total BAL cells in addition to a significant increase in neutrophils as compared with sham-exposed animals. Oral administration of dexamethasone did not attenuate total BAL cell or neutrophil counts. However, administration of an oral PDE4 inhibitor, significantly reduced percent neutrophil and macrophage counts. To assess expression of genes involved in this model, transcriptional profiling was performed on whole lungs harvested on day 4. Genes with the greatest fold increase included SAA3, CXCL1, 2 and 5 and NOXO1. In addition, multiplex analysis of BALF collected on day 4, revealed significant elevation in inflammatory mediators including cytokines, chemokines and metalloproteinases. Matrix metalloproteinase (MMP)–9 and –12 knockout (KO) mice were also tested in this model to evaluate whether or not these deficiencies influence acute smoke-induced inflammation. Total BAL inflammation and neutrophil counts were decreased in smoke exposed MMP-9 and -12 KO mice compared to wild type controls. These effects, however seem to be strain dependent as they were noted only in BALB/c KO animals. Deficiencies in MMP-9 and –12 appear to be beneficial in having an anti-inflammatory effect following acute exposure to smoke.

